# High leafy and root vegetables and high rice dietary patterns were associated with primary and secondary bile acid levels in the feces

**DOI:** 10.1038/s41598-025-86273-8

**Published:** 2025-01-15

**Authors:** Yosuke Saito, Toyoaki Sagae

**Affiliations:** 1 Department of Clinical Nutrition, Faculty of Health and Wellness Sciences, Hiroshima International University, 5-1-1, Hirokoshingai, Kure, Hiroshima, 737−0112 Japan; 2 Department of Human Life Sciences, Sakura No Seibo Junior College, 3-6 Hanazono-Cho, Fukushima-Shi, Fukushima, 960-8585 Japan; 3 Department of Health and Nutrition, Yamagata Prefectural Yonezawa University of Nutrition Sciences, 6-15-1, Torimachi, Yonezawa, Yamagata, 992−0025 Japan

**Keywords:** Colorectal cancer prevention, Fecal bile acids, Dietary patterns, Reduced-rank regression, Rice, Vegetable, Nutrition, Preventive medicine

## Abstract

Colorectal cancer has the second highest mortality among cancer sites worldwide, with increasing morbidity, high recurrence rates, and even poorer postoperative quality of life. Therefore, preventive strategies for colorectal cancer should be established. This study aimed to cross-sectionally explore dietary patterns affecting the intestinal metabolism of bile acids (BAs), a risk factor for colorectal cancer, in young Japanese women. We collected fecal samples for intestinal microbiota and BA analysis. We used the Bristol scale to determine 1-week defecation status. Moreover, the brief-type self-administered diet history questionnaire was used for habitual dietary intake status. Reduced-rank regression analysis revealed dietary patterns related to fecal BA levels. The relationship between dietary patterns and fecal BA levels was adjusted for defecation status and intestinal microbiota variables using analysis of covariance. Reduced-rank regression analysis generated two dietary pattern scores related to fecal BA levels. First, the score was associated with a greater intake of leafy and root vegetables, and higher values were associated with greater fecal cholic and chenodeoxycholic acid levels and lower deoxycholic and lithocholic acid levels. Second, the score was associated with greater rice intake and lower Western sweets, pork, beef, and egg intake, and higher values were associated with lower deoxycholic and lithocholic acid levels. These relationships remained after adjusting for intestinal microbiota and defecation status variables.

## Introduction

Colorectal cancer is the third most common cancer worldwide, and its morbidity and mortality may increase in the future^1^. Furthermore, the recurrence rate of colorectal cancer is high^2^, and colorectal cancer surgery negatively affects the quality of life of patients^3^. Therefore, preventive strategies for colorectal cancer should be developed^4–6^. The morbidity of colorectal cancer shows marked geographic differences, which may be due to differences in diet^7^. However, most nutrients in the diet are absorbed in the small intestine and do not pass into the large intestine. Bile acids (BAs) are promising candidates that can explain the relationship between diet and colorectal cancer risk^8^.

Cholic acid (CA) and chenodeoxycholic acid (CDCA), primary BAs synthesized from cholesterol in the liver, are conjugated by glycine or taurine and then secreted into the intestinal tract, where they play a role in solubilizing dietary fats. Intestinal bacteria gradually modify some conjugated primary BAs secreted into the intestinal tract, although many are reabsorbed in the terminal ileum and returned to the liver (enterohepatic circulation). First, microbial bile salt hydrolases deconjugate conjugated primary BAs to free forms, which are hydrophobic and have low reabsorption rates at the terminal ileum. Subsequently, free primary BAs are further 7α-dehydroxylated by microbial enzymes, and CA and CDCA are converted to secondary BAs, deoxycholic acid (DCA) and lithocholic acid (LCA), respectively. Most LCAs are not reabsorbed and excreted in the feces, but some DCAs are reabsorbed, transported to the liver, and accumulated in the bile pool^9^.

DCAs and LCAs possess tumor-promoting activity and are considered damaging BAs^10^. Fecal DCA levels were elevated in patients with colorectal cancer and individuals at high risk of colorectal cancer^11^. In addition, BAs act as signaling molecules^12^. TGR5, a G-protein–coupled BA receptor 1 expressed within the gastrointestinal tract, strongly responds to DCA and LCA and is involved in BA metabolism and intestinal motor regulation. Farnesoid X receptors (FXRs) strongly respond to CDCA and regulate BA synthesis. Decreased expression and activation of TGR5 and FXR are associated with increased cytotoxicity, inflammation, and secondary BA production^13^. Alterations in BA composition in the intestinal tract may affect TGR5 and FXR activation and cause intestinal inflammatory response dysregulation, and persistent inflammation may induce colorectal cancer^14^. Thus, BA metabolism regulation in the intestinal tract may prevent the development and recurrence of colorectal cancer.

Long-term dietary changes may be a promising strategy to reduce colorectal cancer risk^5,6,15^. The effects of fat and fiber on intestinal BA metabolism may vary depending on the source of intake of these nutrients^16^. Although animal fat intake is associated with colorectal cancer risk, polyunsaturated fatty acid intake in fish may reduce this risk^17^. Therefore, the effect of diet on intestinal BA metabolism should be evaluated on a food basis rather than on a nutrient basis.

Recent nutritional epidemiology studies have focused on dietary patterns rather than single nutrients because of the complex relationship between disease risk and dietary intake^18^. Reduced-rank regression (RRR) is a dietary pattern analysis tool that uses a disease-related variable (biomarker or nutrient) as an intermediate response variable and generates a dietary pattern score that can explain this variable adequately^19^. The RRR can be used to explore dietary patterns that better predict disease risk and may provide necessary information for disease prevention strategies^20^.

The extreme differences in dietary habits between countries have resulted in marked differences in intestinal BA metabolism^11^. However, only a few studies in populations with similar dietary habits (same region, age, and sex) have evaluated the relationship between diet and intestinal BA metabolism. Whether relatively small dietary changes can alter BA metabolism in the intestinal tract remains unclear. This study aimed to explore dietary patterns affecting intestinal BA metabolism using RRR in young female Japanese participants.

## Methods

### Study participants

Participants were recruited from the Yamagata Prefectural Yonezawa University of Nutrition Sciences and Sakura no Seibo Junior College. The inclusion criteria were as follows: (1) aged 18–29 years, (2) not previously diagnosed with hypertension, diabetes, dyslipidemia, or other chronic disease, (3) not currently receiving treatment for gastrointestinal disease, and (4) not habitually taking medications for diarrhea or constipation. Five male participants who volunteered for this study were excluded from the analysis to control for the effects of confounding factors. To assess the natural defecation status and collect fecal samples, we excluded two participants who received diarrheal or constipation medication 1 week before fecal sampling. Ultimately, we included 63 participants. Written informed consent was obtained from all participants. The Ethics Committee of the Yamagata Prefectural Yonezawa University of Nutrition Sciences approved our study protocol (Approval No. 2019–9), which followed the principles of the Declaration of Helsinki.

## Protocol

The participants underwent three examinations: (A) recording of defecation status and medication intake 1 week before fecal collection, (B) fecal collection, and (C) administration of questionnaires on habitual diet (brief-type self-administered diet history questionnaire [BDHQ])^21^. Throughout the study period, the participants were instructed to live normally without any restrictions on physical activity and dietary intake. The study was conducted without considering the menstrual period of the participants as a factor.

(A) Defecation status records

The participants recorded their defecation status using the Bristol Stool Form Scale (BSFS) for 1 week before fecal collection. The BSFS is a tool designed to classify fecal form into seven categories, with types 1–2 and 6–7 being hard and watery feces, respectively, and is widely used in clinical and research fields^22^. The participants were fully instructed regarding the BSFS, and they were asked to self-assess and record the fecal form immediately after defecation. The participants also recorded all medications administered over this period, and they maintained these records until the feces were collected.

(B) Collection and analysis of feces

The participants collected their feces at a convenient time after recording their defecation status for 1 week. The feces were collected at once in a special fecal collection cup, stirred several times with a spoon, placed in a container, which was then placed in a cool bag with a coolant, and submitted to the researcher as soon as possible (i.e., samples collected in the early morning on weekdays were submitted in the morning; samples collected on campus were submitted promptly; if the samples were collected on a holiday or at night, the researcher picked up the sample at the participant’s home). The participants also evaluated their collected feces using the BSFS. The submitted fecal samples were stored at − 80 °C until use.

Fecal BA concentrations were analyzed with Techno Suruga Laboratory Co., Ltd., (Shizuoka, Japan) using liquid chromatography in combination with hybrid quadrupole time-of-flight mass spectrometry (LC-QTOF-MS). BAs were extracted from fecal samples using a previously described method^23^ with slight modifications. Subsequently, 100 mg of fecal sample was suspended in 0.9 mL of sodium acetate buffer (100 mM, pH 5.6) mixed with ethanol in a 2-mL tube with zirconia beads and then heat-treated at 85 °C for 30 min. After centrifugation at 18,400 × *g* for 10 min, the supernatant was diluted four-fold with water and subjected to solid-phase extraction using a Bond Elut C18 cartridge (Agilent Technologies, USA). The solvent of the obtained extract was evaporated, and the residue was dissolved in 50% ethanol with an internal standard. This solution was filtered through a hydrophilic polytetrafluoroethylene filter and used as a sample for LC-QTOF-MS analysis. We used an LC-QTOF-MS instrument consisting of Waters ACQUITY UPLC, Xevo G2-S QTOF, and an electrospray ionization probe (Waters, USA). An Acquity UPLC BEH C18 column (1.7 µm, 2.1 mm × 150 mm; Waters, USA) was used at 65 °C. Separation was performed via gradient elution using 0.1% formic acid aqueous solution (solvent A) and acetonitrile containing 0.1% formic acid (solvent B) at a flow rate of 0.5 mL/min. The gradient elution program for solvent B was as follows: 0 − 0.5 min, 30%; 0.5–1.0 min, 30%–35%; 1.0–7.0 min, 35%–40%; 7.0–10.0 min, 40%–50%; 10.0–11.5 min, 50%–95%; and 11.5–13.0 min, 95%. The QTOF mass spectrometer was operated in negative ion mode. The desolvation and collision gases were nitrogen and argon, respectively. We used the following parameters: capillary voltage, 0.5 kV; sampling cone voltage, 20 V; source temperature, 150 °C; desolvation temperature, 450 °C; cone gas flow, 100 L/h; desolvation gas flow, 1000 L/h; scan time, 0.3 s; data acquisition region, 50–850 m/z. Leucine enkephalin was used as the lock mass, which generated a 554.2615 Da [M–H] − ion.

We analyzed the fecal microbiota with Techno Suruga Laboratory Co., Ltd. (Shizuoka, Japan) by using terminal restriction fragment length polymorphism (T-RFLP) targeting bacterial 16S rDNA. We extracted DNA from fecal samples following a previously published protocol^24^. We suspended 100 mg of each fecal sample in a mixture of 4 M guanidine thiocyanate, 100 mM Tris–HCl (pH 9.0), and 40 mM EDTA, followed by beating with zirconia beads using a FastPrep-24 5G instrument (MP Biomedicals, USA) to obtain crude extracted DNA. An automated DNA isolation system (GENE PREP STAR PI-480, Kurabo Industries, Japan) and a DNA isolation reagent kit (NR-201, Kurabo Industries, Japan) were used to purify the crude DNA. The DNA concentration was estimated via spectrophotometry using a NanoDrop ND8000 (Thermo Fisher Scientific, USA), and the final concentration of the DNA sample was adjusted to 10 ng/μL. We amplified 16S rDNA, performed restricted enzyme digestion, and conducted fragment analysis based on a previously published protocol^25,26^. The 16S rDNA was amplified using a fluorescently labeled 516f. primer (5′-TGCCAGCAGCCGCGGTA-3′) and 1510r primer (5′-GGTTACCTTGTTACGACTT-3′). The resulting 16S rDNA amplicons were digested using FastDigest BselLI (BslI, Thermo Fisher Scientific, USA) for 10 min, and the digestion products were used for fragment analysis using the ABI PRISM 3130 xl Genetic Analyzer System (Applied Biosystems, USA). The taxa included in Clostridiales were classified based on the *Clostridium*cluster^27,28^.

(C) Assessment of habitual diet

The BDHQ was used to assess habitual diet during the preceding month^21^. It included questions on the frequency of consumption of 58 foods and beverages as well as amount per occasion of alcohol consumption. Nutrient intake was calculated using a commercial computer algorithm for the BDHQ, which was primarily based on the Standard Table of Food Composition in Japan^29^. Participants who consumed alcohol > 3 days per week and > 20 g of ethanol per occasion were defined as habitual drinkers. The BDHQ has a satisfactory ranking ability for energy-adjusted nutrient intake^30^. Therefore, nutrient and food item intakes were energy-adjusted using the energy density model and expressed as density (per 1000 kcal of energy intake).

## Generating dietary pattern scores using RRR

RRR is a useful statistical method that can generate dietary patterns based on clinical outcomes (intermediate response variable)^19,31^. In the current study, it was used to generate habitual dietary patterns that influence fecal BA levels. Some dietary pattern studies based on RRR have used known disease risk-related nutrients as intermediate response variables^32,33^. As we aimed to explore unknown diet-related factors associated with intestinal BA metabolism, we used the nutrients that were significantly correlated with fecal BA levels as the intermediate response variable. Other studies have used the same approach^34^. After energy adjustment using the density method (g/1000 kcal), the 53 food and beverage intakes estimated based on the BDHQ were used as explanatory variables. Random sample cross-validation and subsequent van der Voet’s test were used to define the optimal number of dietary patterns. RRR was performed using the statistical software package SAS® OnDemand for Academics (SAS Institute Inc., Cary, North Carolina, United States).

## Statistical analysis

The distributions of fecal BA levels were right-skewed; hence, natural log transformation was applied to make the distribution more symmetric. The Shapiro–Wilk test was used to assess the normality of our dataset. Relationships between normally distributed variables were evaluated using Pearson’s correlation, whereas variables that were not normally distributed were analyzed using Spearman’s correlation. We assessed differences among the three groups based on dietary pattern scores using one-way analysis of variance. If Levene’s test showed homogeneity of variance, we used Tukey’s test for post hoc pairwise multiple comparisons. Games–Howell test was used for samples with nonhomogeneous variances. Data were presented as means ± standard deviation. Analysis of covariance (ANCOVA plus Sidak post hoc test) was used to evaluate the relationship between dietary patterns and fecal BA levels, excluding the effects of defecation status and intestinal microbiota. All statistical analyses, except for RRR, were performed using the Statistical Package for the Social Sciences software ver. 28.0 for Windows (IBM SPSS, Inc., Chicago, IL, USA). *p*-values of < 0.05 were considered to indicate statistical significance.

## Covariates

As defecation status and intestinal microbiota influence intestinal BA metabolism, the relationships between dietary patterns and fecal BA levels were adjusted for these variables. The variables of defecation status included the frequencies of hard, normal, and watery stools (times/week). We selected the relative abundances of the following genera containing bacteria involved in intestinal BA metabolism as covariates: *Bifidobacterium*, *Lactobacillales* (Order), *Bacteroides*, and *Clostridium*subcluster XIVa^35–38^. The variables of defecation status and intestinal microbiota were dimensionally reduced based on principal component analysis. Subsequently, the four principal components generated were used as covariates (Supplemental Table 1 and 2). The dimensionality reduction was attributed to the variables being interrelated, and analysis in a relatively small sample size requires restriction of the number of covariates^39^.

## Results

### Participant characteristics

Table 1 shows the participant characteristics. All participants were female, with a mean age of 19.9 ± 0.9 years. The mean body mass index (BMI) was 21.3 kg/m^2^, and the majority (79%) of the participants had normal body weight (18.5 ≤ BMI < 25 kg/m^2^). The mean total BA concentration in the feces was 4.21 µmol/g, with the majority of BAs being CA (15.9%), CDCA (10.2%), DCA (38.2%), and LCA (21.1%). The mean frequency of defecation was 8.6 times/week, and 74.4% of the participants had normal feces. Five participants were habitual drinkers.

**Table 1:** Characteristics of young female participants in this study.

All participants (n = 63)	
Age (year)	19.9 ± 0.9
Female (%)	100
Height (cm)	157 ± 5
Body weight (kg)	52.5 ± 7.5
Body mass index (kg/m^2^)	21.3 ± 2.6
Fecal bile acid levels (μmol/g) ^a^	
Total bile acids	4.21 ± 3.78
DCA	1.61 ± 1.48
LCA	0.89 ± 0.98
CA	0.67 ± 1.15
CDCA	0.43 ± 0.96
UDCA	0.22 ± 0.37
7-oxo-DCA	0.11 ± 0.23
7-oxo-LCA	0.07 ± 0.42
Frequency of defecation (time/week)	8.6 ± 4.0
Hard feces (BSFS type 1–2)	1.7 ± 2.5
Normal feces (BSFS type 3–5)	6.4 ± 3.7
Watery feces (BSFS type 6–7)	0.5 ± 1.2
Microbiota (%)	
*Bifidobacterium*	18.9 ± 12.7
*Lactobacillales* (Order)	7.6 ± 6.3
*Bacteroides*	30.5 ± 13.8
*Prevotella*	0.2 ± 1.5
*Clostridium* cluster IV	6.6 ± 4.2
*Clostridium* subcluster XIVa	22.6 ± 11.5
*Clostridium* cluster IX	3.9 ± 5.2
*Clostridium* cluster XI	0.6 ± 1.0
*Clostridium* cluster XVIII	1.1 ± 2.3
others	8.0 ± 5.1
Habitual diet	
Protein (g/1000 kcal)	36.7 ± 5.1
Fat (g/1000 kcal)	31.2 ± 5.3
Cholesterol (mg/1000 kcal)	221 ± 72
Total dietary fiber (g/1000 kcal)	6.1 ± 1.4
Soluble dietary fiber (g/1000 kcal)	1.6 ± 0.4
Insoluble dietary fiber (g/1000 kcal)	4.4 ± 0.9
Sodium (mg/1000 kcal)	2147 ± 387
Potassium (mg/1000 kcal)	1228 ± 250
Calcium (mg/1000 kcal)	240 ± 63

Data presented as % or mean ± standard deviation. Abbreviations: BSFS, Bristol Stool Form Scale; CA, cholic acid; CDCA, chenodeoxycholic acid; DCA, deoxycholic acid; LCA, lithocholic acid; UDCA, ursodeoxycholic acid. ^a^ Bile acid levels were measured per fresh fecal mass.

### Correlation between fecal BA levels and nutrient intake

**Fig. 1:**
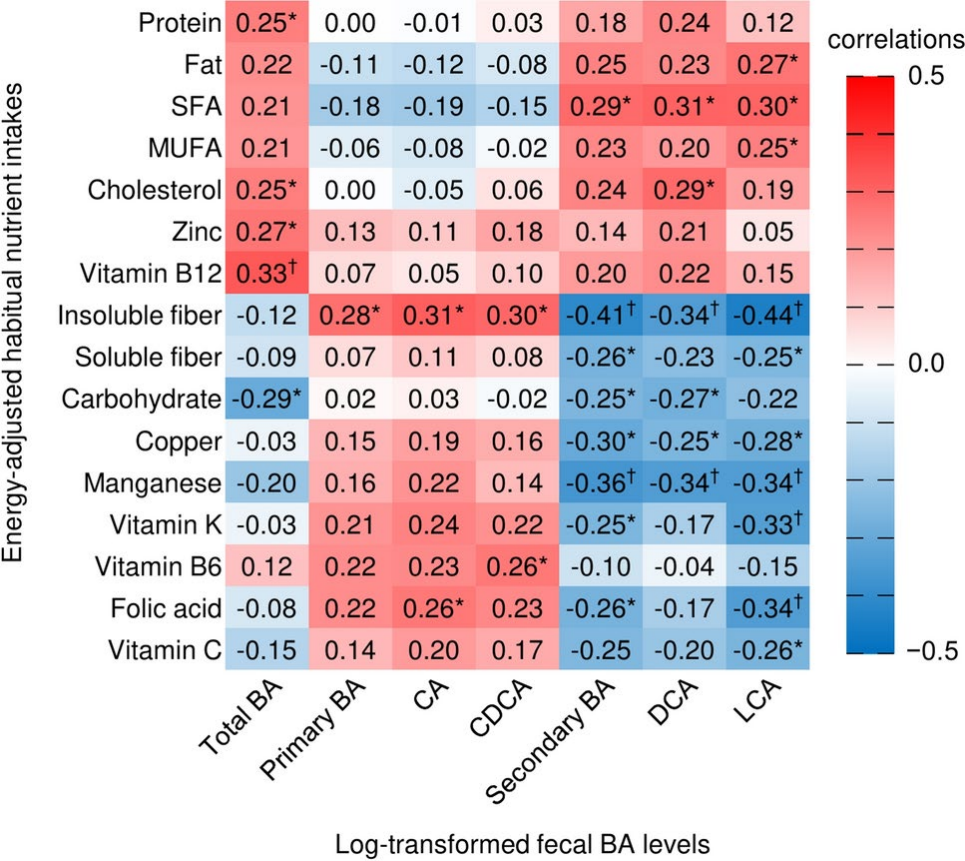
Spearman correlation coefficients between fecal BA levels and habitual nutrient intake in young female participants. Primary BAs were CA + CDCA. Secondary BAs were DCA + LCA. Abbreviations: BA, bile acid; CA, cholic acid; CDCA, chenodeoxycholic acid; DCA, deoxycholic acid; LCA, lithocholic acid; SFA, Saturated fatty acids; MUFA, Monounsaturated fatty acid. *p < 0.05, ^†^*p* < 0.001.

Figure 1 shows Spearman’s correlation coefficients between fecal BA levels and habitual nutrient intake. CA and CDCA were significantly positively correlated with insoluble fiber (r = 0.314; *p* = 0.012 and r = 0.298; *p* = 0.018, respectively). Conversely, DCA and LCA showed significant positive correlations with saturated fatty acids (r = 0.306; *p* = 0.015 and r = 0.302; *p* = 0.016, respectively) and significant negative correlations with insoluble fiber (r = − 0.338; *p* = 0.007 and r = − 0.440; *p* < 0.001, respectively), copper (r = − 0.253; *p* = 0.046 and r = − 0.278; *p* = 0.027, respectively), and manganese (r = − 0.341; *p* = 0.006 and r = − 0.339; *p* = 0.007, respectively).

### Dietary patterns related to BAs

Fecal CA levels were strongly correlated with CDCA levels (r = 0.896; *p* < 0.001, data not shown). Fecal DCA levels were strongly correlated with LCA levels (r = 0.721; *p* < 0.001, data not shown). Furthermore, primary BA (CA + CDCA) levels were negatively correlated with secondary BA (DCA + LCA) levels (r = − 0.441; *p* < 0.001, data not shown). Therefore, the RRR was used to explore dietary patterns related to primary and secondary BA levels. To explore dietary patterns related to primary BAs, we used insoluble fiber, which was significantly correlated with primary BAs, as a response variable. To explore dietary patterns related to secondary BAs, we used the following intermediate response variables: insoluble fiber, soluble fiber, vitamin K, folic acid, copper, manganese, carbohydrates, and saturated fatty acids.

Table 2 shows the contribution ratio of dietary pattern scores to the response and explanatory variables and the correlation coefficient between dietary pattern scores and habitual nutrient intake. One dietary pattern score for primary BAs was generated (dietary pattern [DP]−1), with a 100% contribution to the response variable. Four dietary pattern scores for secondary BAs were generated (DP-2, 3, 4, and 5). The contribution ratio of DP-2 to the response variable was 48%. We excluded DP-2 because it was very similar to DP-1 (Pearson’s correlation, r = 0.942; *p* < 0.001, data not shown). DP-3 had a 26.1% contribution to the response variable, with a strong positive correlation with carbohydrates and a strong negative correlation with saturated fatty acids. We excluded DP-4 and 5 due to their low contribution to the response variable. Finally, DP-1 and DP-3 were used for further analysis.

**Table 2:** Contribution ratio of dietary pattern scores generated by reduced-rank regression to response and explanatory variables and correlation coefficient between dietary pattern scores and habitual nutrient intake.

	Dietary pattern for primary BAs	Dietary pattern for secondary BAs			
	DP-1	DP-2	DP-3	DP-4	DP-5
Contribution ratio					
to response variable (nutrients, %)	100.0	48.0	26.1	12.6	4.8
to explanatory variables (foods, %)	5.5	5.1	5.3	3.9	4.3
Pearson’s correlation coefficient					
Insoluble fiber	1.000^†^	0.942^†^	0.055	− 0.083	− 0.126
Soluble fiber	0.876^†^	0.905^†^	− 0.131	− 0.159	0.062
Vitamin K	0.761^†^	0.867^†^	− 0.151	− 0.144	− 0.173
Folic acid	0.770^†^	0.852^†^	− 0.200	0.371*	− 0.196
Copper	0.659^†^	0.739^†^	0.355^†^	− 0.288*	0.463^†^
Manganese	0.210	0.299^†^	0.404^†^	0.840^†^	0.167
Carbohydrate	− 0.024	− 0.107	0.937^†^	− 0.059	− 0.084
Saturated fatty acids	− 0.162	− 0.093	− 0.913^†^	0.160	0.223

DP-1 was generated using insoluble fiber as a response variable. DP-2, 3, 4, and 5 were generated using insoluble fiber, soluble fiber, vitamin K, folic acid, copper, manganese, carbohydrate, and saturated fatty acids as a response variable. Abbreviations: DP, dietary pattern; DCA, deoxycholic acid; LCA, lithocholic acid; CA, cholic acid; CDCA, chenodeoxycholic acid.

*p < 0.05 ^†^*p* < 0.001.

**Fig. 2:**
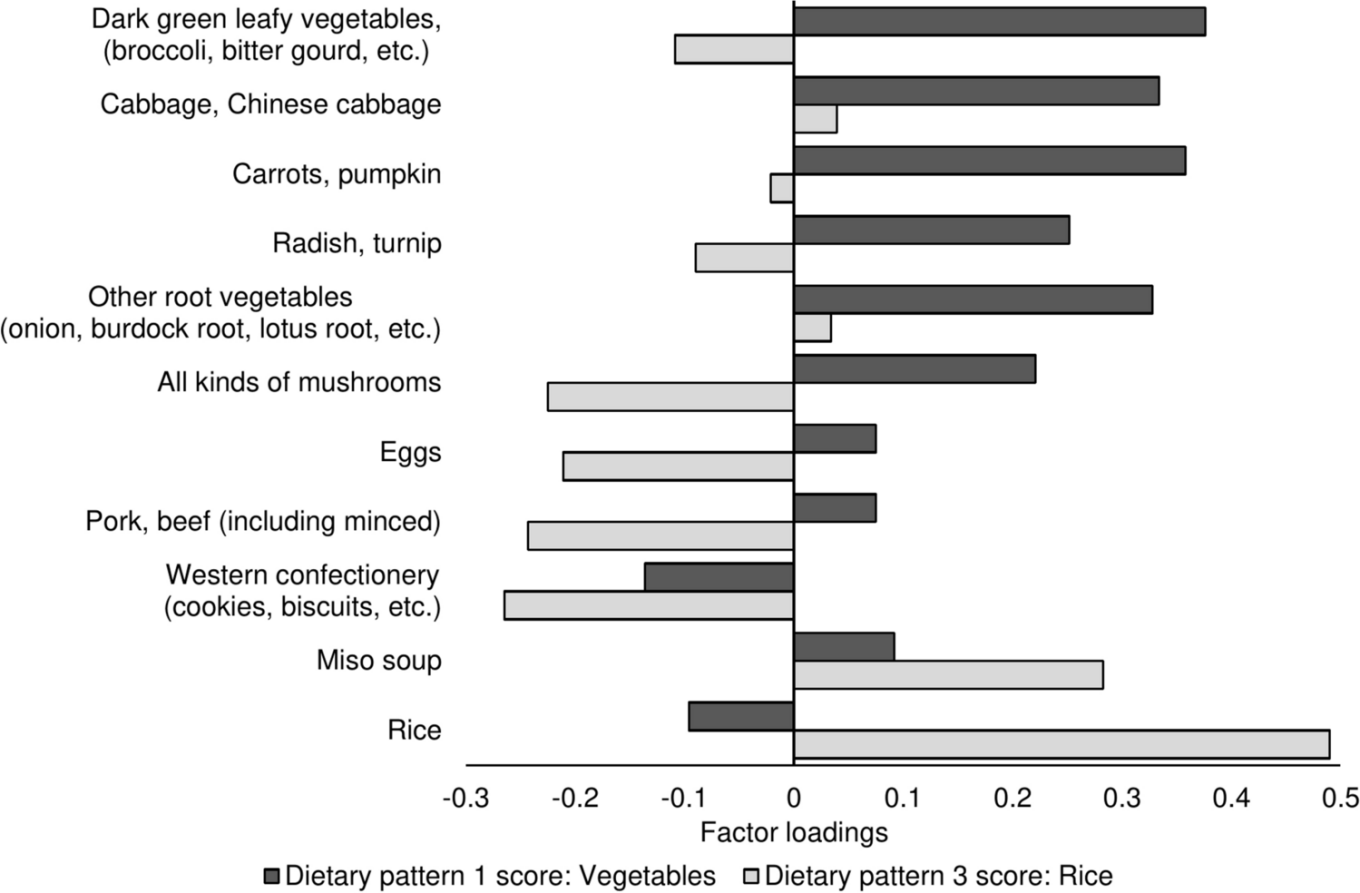
Factor loadings of major food items on the dietary pattern scores generated by reduced-rank regression.

Figure 2 shows factor loadings for major food items on DP-1 and 3 scores. DP-1 was associated with a high intake of dark green leafy vegetables, cabbage, Chinese cabbage, carrots, pumpkin, radish, turnip, other root vegetables, and all kinds of mushrooms and was associated with vegetable-related dietary pattern scores. DP-3 was associated with a high intake of rice and miso soup and a low intake of Western confectionery, pork, beef, eggs, and all kinds of mushrooms, which was attributed to a high rice intake. Supplemental Table 3 shows the factor loadings of the 53 food items on the DP-1–5 scores.

### Relationships between dietary patterns and defecation status and intestinal microflora

We divided the study participants into tertiles based on the DP-1 and DP-3 scores and assessed the differences between tertiles in defecation status, intestinal microbiota, and habitual diet (Table 3). The frequency of normal stools in tertile 3 (high) for DP-1 was significantly greater than that in tertile 1 (low). The intestinal microbiota was not significantly different in the DP-1 tertile. The defecation status and intestinal microbiota were not significantly different between the DP-3 tertiles.

**Table 3:** Defecation frequency, intestinal microbiota, and habitual nutrient intake of young female participants in this study disaggregated by the dietary pattern 1 and 3 scores.

	Dietary pattern 1 scores: Vegetables			*p*	Dietary pattern 3 scores: Rice			*p*
	Tertile 1 (low)	Tertile 2	Tertile 3 (high)		Tertile 1 (low)	Tertile 2	Tertile 3 (high)	
N	21	21	21		21	21	21	
Female (%)	100	100	100		100	100	100	
Body mass index (kg/m^2^)	20.8 ± 2.5	20.9 ± 1.9	22.3 ± 3.2	0.116	21.7 ± 3.0	20.7 ± 2.2	21.6 ± 2.6	0.391
Frequency of defecation (time/week)	6.9 ± 3.0	8.9 ± 2.5	10.0 ± 5.4	0.031	9.4 ± 3.5	7.8 ± 3.8	8.7 ± 4.6	0.428
Hard feces (BSFS type 1–2)	1.5 ± 2.1	1.8 ± 2.0	1.8 ± 3.3	0.925	2.2 ± 2.8	1.1 ± 1.8	1.7 ± 2.7	0.363
Normal feces (BSFS type 3–5)	4.7 ± 2.7 ^a^	6.7 ± 3.3 ^ab^	7.7 ± 4.4 ^b^	0.025	6.3 ± 3.7	6.2 ± 4.2	6.6 ± 3.3	0.946
Watery feces (BSFS type 6–7)	0.7 ± 1.5	0.4 ± 0.6	0.5 ± 1.4	0.820	0.8 ± 1.8	0.4 ± 0.6	0.4 ± 0.9	0.464
Microbiota (%)								
*Bifidobacterium*	20.5 ± 14.9	17.6 ± 11.6	18.5 ± 11.8	0.750	20.3 ± 14.4	15.5 ± 12.1	20.9 ± 11.2	0.323
*Lactobacillales* (Order)	7.5 ± 7.0	8.3 ± 5.9	6.9 ± 6.2	0.771	7.8 ± 6.5	7.8 ± 7.0	7.1 ± 5.5	0.907
*Bacteroides*	31.9 ± 14.0	29.5 ± 11.1	30.1 ± 16.4	0.843	27.3 ± 16.5	33.4 ± 11.6	30.8 ± 12.9	0.362
*Prevotella*	0.0 ± 0.0	0.1 ± 0.5	0.6 ± 2.6		0.1 ± 0.3	0.0 ± 0.0	0.7 ± 2.6	
*Clostridium* cluster IV	7.8 ± 5.1	5.4 ± 3.2	6.6 ± 4.1	0.174	6.5 ± 4.5	6.7 ± 4.0	6.7 ± 4.5	0.988
*Clostridium* subcluster XIVa	20.5 ± 10.3	25.1 ± 12.8	22.1 ± 11.3	0.421	23.3 ± 14.0	24.0 ± 9.7	20.5 ± 10.6	0.589
*Clostridium* cluster IX	3.5 ± 4.3	3.5 ± 5.1	4.7 ± 6.2	0.726	4.3 ± 5.7	3.0 ± 5.6	4.4 ± 4.2	0.636
*Clostridium* cluster XI	0.5 ± 0.6	0.8 ± 1.6	0.4 ± 0.7	0.546	0.5 ± 0.8	0.9 ± 1.6	0.4 ± 0.5	0.326
*Clostridium* cluster XVIII	0.7 ± 0.9	1.7 ± 3.5	1.0 ± 1.7	0.408	0.7 ± 0.9	1.4 ± 3.4	1.3 ± 1.9	0.593
others	6.9 ± 5.2	8.0 ± 3.6	9.0 ± 6.3	0.418	9.2 ± 6.5	7.3 ± 3.8	7.4 ± 4.6	0.395
Habitual diet								
Protein (g/1000 kcal)	35.0 ± 4.2 ^a^	36.0 ± 5.1 ^ab^	39.0 ± 5.1 ^b^	0.023	40.0 ± 4.8 ^a^	36.7 ± 3.8 ^b^	33.3 ± 4.3 ^c^	< .001
Fat (g/1000 kcal)	31.9 ± 4.9	29.5 ± 5.9	32.2 ± 4.9	0.199	36.4 ± 2.8 ^a^	31.2 ± 2.6 ^b^	26.0 ± 4.0 ^c^	< .001
Saturated fatty acids (g/1000 kcal)	8.8 ± 1.8	7.9 ± 1.8	8.4 ± 1.9	0.261	10.1 ± 1.2 ^a^	8.6 ± 0.9 ^b^	6.4 ± 1.2 ^c^	< .001
Cholesterol (mg/1000 kcal)	215 ± 72	215 ± 69	234 ± 76	0.619	266 ± 75 ^a^	227 ± 44 ^ab^	171 ± 60 ^b^	< .001
Carbohydrate (g/1000 kcal)	136 ± 14	138 ± 19	133 ± 16	0.569	120 ± 8 ^a^	134 ± 7 ^b^	153 ± 12 ^c^	< .001
Total dietary fiber (g/1000 kcal)	4.6 ± 0.7 ^a^	6.1 ± 0.4 ^b^	7.7 ± 0.6 ^c^	< .001	6.3 ± 1.4	6.0 ± 1.1	6.2 ± 1.5	0.708
Soluble dietary fiber (g/1000 kcal)	1.2 ± 0.3 ^a^	1.6 ± 0.2 ^b^	2.0 ± 0.3 ^c^	< .001	1.7 ± 0.4	1.6 ± 0.4	1.5 ± 0.5	0.476
Insoluble dietary fiber (g/1000 kcal)	3.4 ± 0.5 ^a^	4.4 ± 0.2 ^b^	5.4 ± 0.5 ^c^	< .001	4.5 ± 1.0	4.2 ± 0.7	4.5 ± 1.0	0.563

DP, dietary pattern; BSFS, Bristol Stool Form Scale. *Bile acid levels were measured per fresh fecal mass. ^abc^ Different letters indicate statistically significant differences between the groups (Tukey’s post hoc test or Games − Howell test, *p* < 0.05).

### Relationship between dietary patterns and fecal BA levels

**Fig. 3:**
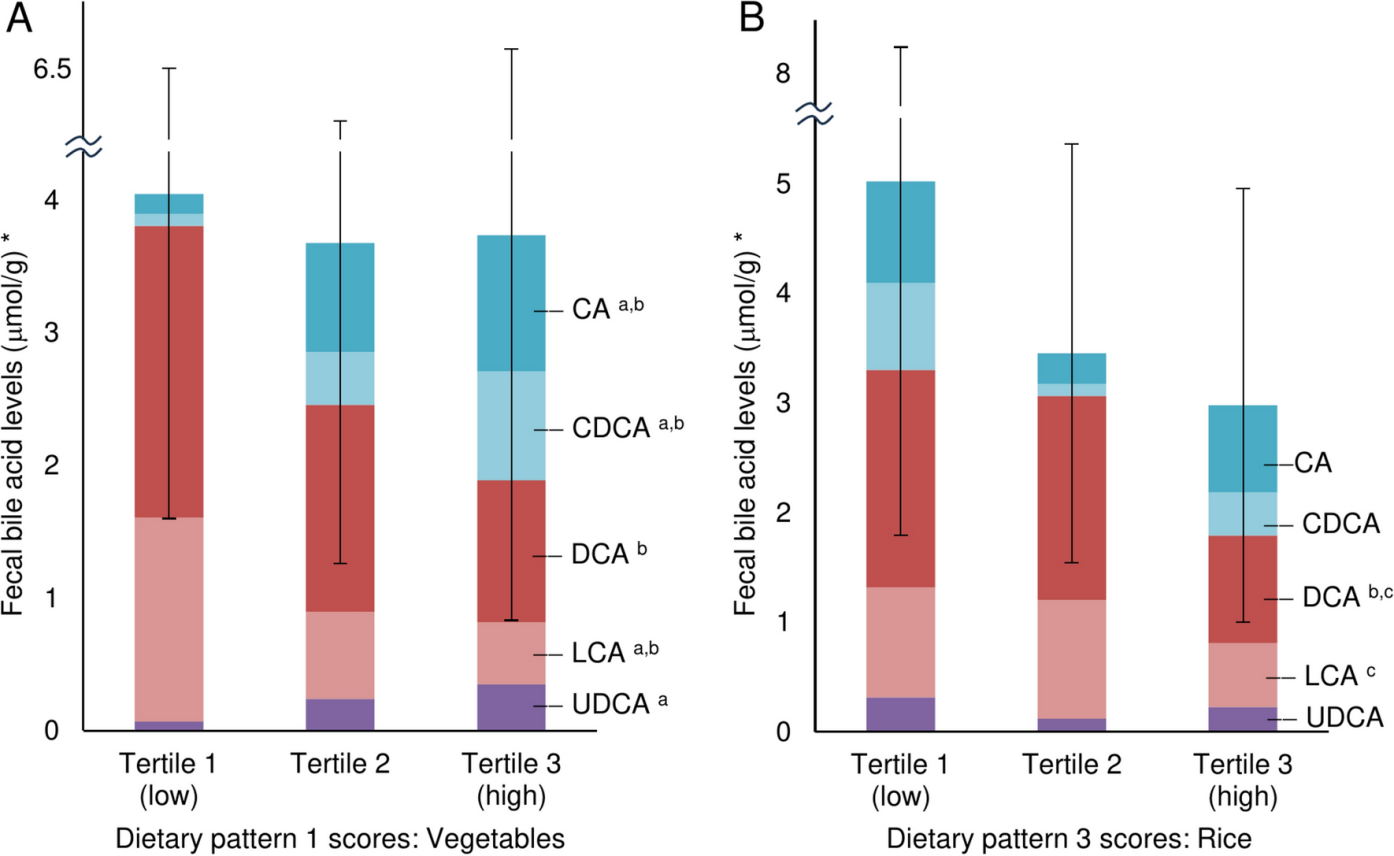
Fecal BA levels of young female participants disaggregated by dietary pattern 1 (A) and 3 (B) scores. *BA levels were measured per fresh fecal mass. ^a^ Tukey’s post hoc test or Games − Howell test, *p* < 0.05, Tertile 1 vs. 2. ^b^ Tukey’s post hoc test or Games − Howell test, *p* < 0.05, Tertile 1 vs. 3. ^c^ Tukey’s post hoc test or Games − Howell test, *p* < 0.05, Tertile 2 vs. 3. Abbreviations: BA, bile acid; CA, cholic acid; CDCA, chenodeoxycholic acid; DCA, deoxycholic acid; LCA, lithocholic acid; UDCA, ursodeoxycholic acid.

Figure 3 shows the differences in fecal BA levels between DP-1 and DP-3 tertiles. Fecal CA and CDCA levels were significantly greater as the DP-1 score increased. Conversely, the DCA and LCA were significantly lower. Furthermore, the fecal DCA levels in tertile 3 (high) for DP-3 were significantly lower than those in tertiles 1 (low) and 2.

Differences in fecal BA levels between the dietary pattern score tertiles were adjusted for variables of defecation status and intestinal microbiota using ANCOVA (Table 4.). Fecal primary BA levels were significantly greater in tertile 3 (high) for DP-1 than in tertile 1 (low), even after adjusting for defecation status and intestinal microbiota. Fecal secondary BA levels for DP-1 and DP-3 were significantly lower in tertile 3 (high) than in tertile 1 (low), even after adjusting for defecation status and intestinal microbiota. Supplemental Tables 4 and 5 show the relationships between total fecal BA, CA, CDCA, DCA, and LCA levels and DP-1 and DP-3 scores.

**Table 4.** Comparison of fecal BA levels between tertiles based on dietary pattern 1 and 3 scores

		N	Crude		Adjusted *	
			Mean ^†^	95% CI	Mean ^†^	95% CI
Primary BA (mmol/g) ^‡^						
DP-1: Vegetables	Tertile 1 (low)	21	0.18 ^a^	(0.00, 0.55)	0.25 ^a^	(0.00, 0.61)
	Tertile 2	21	0.78 ^ab^	(0.36, 1.33)	0.71 ^ab^	(0.33, 1.19)
	Tertile 3 (high)	21	0.99 ^b^	(0.52, 1.61)	0.94 ^b^	(0.52, 1.48)
	*p*		0.021		0.049	
Secondary BA (mmol/g) ^‡^						
DP-1: Vegetables	Tertile 1 (low)	21	3.14 ^a^	(2.21, 4.35)	2.96 ^a^	(2.04, 4.15)
	Tertile 2	21	1.56 ^b^	(0.99, 2.30)	1.61 ^ab^	(1.01, 2.38)
	Tertile 3 (high)	21	1.29 ^b^	(0.77, 1.95)	1.35 ^b^	(0.82, 2.04)
	*p*		0.004		0.019	
DP-3: Rice	Tertile 1 (low)	21	2.33 ^a^	(1.57, 3.30)	2.28 ^a^	(1.55, 3.22)
	Tertile 2	21	2.54 ^a^	(1.74, 3.58)	2.63 ^a^	(1.82, 3.67)
	Tertile 3 (high)	21	1.06 ^b^	(0.59, 1.67)	1.04 ^b^	(0.59, 1.62)
	*p*		0.007		0.004	

Abbreviations: BA, bile acid; CI, confidence interval; CA, cholic acid; CDCA, chenodeoxycholic acid; DCA, deoxycholic acid; LCA, lithocholic acid.

^abc^ Different letters indicate statistically significant differences between the groups (Sidak post hoc test, *p* <0.05).

^‡^ Bile acid levels were measured per fresh fecal mass.

^†^ The values expressed at the geometric mean (95% confidence interval).

Primary BAs were CA + CDCA. Secondary BAs were DCA + LCA. * Adjusted for defecation status (Two principal components generated from the frequency of hard, normal, and watery stools by principal component analysis) and intestinal microbiota (Two principal components generated from the *Bifidobacterium*, *Lactobacillales*, *Bacteroides*, and *Clostridium* subcluster XIVa by principal component analysis).

## Discussion

A habitual diet is associated with colorectal cancer risk, with BAs being one of the most important mediators^8^. Previous studies have assessed the effect of habitual diet on colorectal cancer risk or intestinal BA metabolism by comparing participants with extremely different diets^7,11^. Using a dietary questionnaire and RRR, the current study identified two dietary patterns that may influence BA metabolism in a cohort of young Japanese women. The results indicated that relatively small dietary changes can control intestinal BA metabolism. Furthermore, in this study, lower scores for both dietary patterns were associated with higher fecal levels of toxic secondary BAs. These dietary patterns are associated with a Western diet, which has been previously associated with high fecal secondary BA levels and risk of colorectal cancer^40^.

### Dietary pattern 1: Vegetable intake and intestinal BA metabolism

DP-1 scores were strongly positively correlated with soluble and insoluble fiber intake, and higher DP-1 scores were associated with lower fecal secondary BA levels. The daily consumption of 15 g of inulin or fructo-oligosaccharides for 3 weeks significantly reduced fecal DCA levels^41^. Compared with a low-resistant starch diet, the administration of a high-resistant starch diet for 4 weeks significantly reduced total and secondary BA levels in the feces^42^. Compared with preintervention, the addition of 13–15 g of wheat bran (daily for 8 weeks) to the normal diet significantly reduced the fecal DCA and LCA levels and 7α-dehydroxylase activity^43^.

Interestingly, the DP-1 score was not correlated with total BA levels but was positively correlated with CA and CDCA levels and negatively correlated with DCA and LCA levels, indicating that high vegetable intake may inhibit the bacterial conversion of primary to secondary BAs by intestinal bacteria.

Dietary fiber can affect intestinal BA metabolism by adsorbing BAs and increasing the viscosity of intestinal contents^44^. Naumann et al. recently conducted an in vitro study and showed that the adsorption of BAs to dietary fiber may be hydrophobic^45^. Furthermore, the adsorptive effect of BAs on dietary fiber may primarily involve insoluble rather than soluble fibers^46^. Our study showed that insoluble fiber intake was more strongly correlated with fecal BA levels than soluble fiber intake. If hydrophobic BAs are adsorbed to and aggregated within insoluble dietary fibers in the intestinal tract via hydrophobic effects, intestinal bacteria may not access BAs, inhibiting the conversion of primary BAs to secondary BAs. Studies evaluating BA-binding capacity in vitro have shown that green leafy vegetables, such as kale and spinach, have greater BA-binding capacity than other vegetables^47,48^. Interestingly, the foods with the highest load on the DP-1 score in our study were dark green leafy vegetables.

Although viscosity is primarily a property of soluble fiber^49^, insoluble fiber may also increase viscosity in the intestinal tract^50^. Insoluble fibers can be solubilized by intestinal bacteria in the intestinal tract^51^. Increased viscosity of intestinal contents may decrease the diffusion rate of BAs in the intestinal tract, resulting in the inaccessibility of intestinal bacteria to BAs^52^.

Intestinal bacteria metabolize dietary fibers, converting them into short-chain fatty acids with various beneficial functions^53^. In vitro experiments with mixed fecal bacteria cultures showed that pH reduction by short-chain fatty acids inhibited the conversion of primary BAs to secondary BAs by the bacteria^54^. Compared with a low-fiber diet without oats, the administration of a high-fiber diet containing oats for 6 weeks in rats resulted in a lower pH of intestinal contents, a greater proportion of primary BAs, and a lower proportion of secondary BAs in the feces^55^. Administering resistant starch type 3 in rats increased butyrate levels in the cecum while simultaneously decreasing secondary BA levels^56^.

### Dietary pattern 3: Rice intake and intestinal BA metabolism

DP-3 scores showed a strong positive correlation with carbohydrates and a strong negative correlation with saturated fatty acids, and higher DP-3 scores were associated with significantly lower fecal DCA and LCA levels. Humans and animals showed increased BA secretion and fecal excretion with high lipid intake^57,58^. Dietary intervention trials with animal or plant foods only showed that animal diets significantly increased the gene expression of microbial bile salt hydrolase, which is involved in secondary BA formation^59^.

The traditional Japanese diet includes rice as a staple food, and energy intake from rice accounts for approximately 30% of the total energy intake^60,61^. Rice has a low-fat content (1.8 g/1000 kcal). Conversely, Western sweets, such as cookies, cream puffs, and custard pudding, have high-fat contents (23.1 g, 49.6 g, and 39.7 g/1000 kcal, respectively). Beef thigh, pork thigh, and chicken eggs also have high-fat contents (72.2 g, 55.7 g, and 68.6 g/1000, respectively)^29^. Thus, low DP-3 scores indicate that the source of energy intake has shifted from rice, which has a low-fat and high-carbohydrate content, to high-fat foods. Although miso soup intake contributed to the DP-3 score to some extent, it is usually consumed with rice in Japan^61^.

A large cohort study in Japan showed that rice intake was negatively correlated with meat and egg intake^62^. A cross-sectional study of young Japanese individuals showed that low rice intake frequency was associated with high lipid intake^63^. Matsumoto et al. analyzed trends in food group intake over the past 20 years among young women using data from the Japan’s National Health and Nutrition Examination Survey. They revealed that rice intake has decreased, whereas fat, meat, and confectionery intake has increased^64^. In addition, a study examining food and nutritional consumption patterns among adolescents using data from the UK National Diet and Nutrition Survey showed that a higher confectionery intake was associated with a significantly lower intake of pasta, rice, and other cereals^65^. Thus, high rice intake may lead to low-fat intake, which decreases secondary BA levels in the colonic lumen.

This study had several limitations. First, the cohort was relatively small, and this study only included young Japanese women from two institutions. Hence, some of the results cannot be generalized. Colon motility, prevalence of functional constipation, and intestinal microbiota tend to vary based on age and sex^66–68^. Furthermore, Japanese individuals have a distinct intestinal microbiota^69^. Hence age, sex, and race had to be limited to identifying dietary patterns affecting intestinal BA metabolism in a relatively small cohort. Second, the T-RFLP method could not reveal the role of bacterial species and was inferior to next-generation sequencing in terms of analytical resolution. This may be the reason why there were no significant differences in intestinal microbiota among dietary pattern tertiles in this study. Third, the BDHQ and BSFS scores were self-reported, which may have led to reporting and recall bias. Moreover, young women tend to underreport their dietary intake^70^. Fourth, because RRRs can derive disease-specific dietary patterns and associate them with disease risk, they are less predictive and should be interpreted with caution. Fifth, the study was conducted without considering the menstrual period of the participants. The menstrual cycle may affect colonic transit time^71^, and changes in colonic transit time may result in changes in fecal BA levels^72^. The menstrual cycle may affect fecal BA levels by mediating changes in colonic transit time, and in this study, the number of defecations per week was included as covariates in the ANCOVA. However, it is necessary to investigate whether the menstrual cycle affects intestinal BA metabolism independently of alterations in defecation status. Finally, causal relationships could not be determined because of the cross-sectional study design.

In conclusion, two dietary patterns associated with fecal BA levels were detected in young Japanese women. Dietary patterns with high leafy green and root vegetable intake were associated with lower DCA and LCA levels in the feces and higher CA, CDCA, and UDCA levels. In addition, a shift from high-fat foods, such as meat and Western sweets, to low-fat cereal foods, such as rice, as a source of energy intake was associated with lower fecal DCA and LCA levels. Our study results indicate that relatively small dietary changes can regulate intestinal BA metabolism.

## Additional information

**Clinical Trial Registry: **University Hospital Medical Information Network (UMIN) Center system (UMIN000045639); date of registration: 15/11/2019.

## Supplementary Information

Supplementary Information.

## Data Availability

Deidentified data are available from the corresponding author upon reasonable request.
