# Supplementary material for: High leafy and root vegetables and high rice dietary patterns were associated with primary and secondary bile acid levels in the feces
Source: Sci Rep. 2025 Jan 15;15:2092. doi: 10.1038/s41598-025-86273-8 (PMC11736012; doi:10.1038/s41598-025-86273-8)
Supplement: Supplementary file 1 — Supplementary Information. [file 41598_2025_86273_MOESM1_ESM.pdf]

Supplementary material for ：

High leafy and root vegetables and high rice dietary patterns were associated with primary and secondary bile acid levels in the feces

Yosuke Saito [1,2,*] and Toyoaki Sagae [3]

[1] Department of Clinical Nutrition, Faculty of Health and Wellness Sciences, Hiroshima International University, 5-1-1, Hirokoshingai, Kure, Hiroshima 737−0112, Japan.
[2] Department of Human Life Sciences, Sakura no Seibo Junior College 3-6 Hanazono-cho, Fukushima-shi, Fukushima 960-8585, Japan
[3] Department of Health and Nutrition, Yamagata Prefectural Yonezawa University of Nutrition Sciences 6-15-1, Torimachi, Yonezawa, Yamagata, 992−0025, Japan.
[*] Corresponding author: Yosuke Saito
E-mail: saito-y@hirokoku-u.ac.jp

Supplemental Table 1. The principal components of defecation status generated by principal component analysis, which were used in the analysis of covariance as covariates.

	Principal components of defecation status	
	PC-1	PC-2
Contribution of components (%)	40.1	35.8
Factor loading		
Frequency of hard feces	-0.788	-0.296
Frequency of normal feces	0.761	-0.373
Frequency of watery feces	0.055	0.920

Abbreviations: PC principal components

Supplemental Table 2. The principal components of intestinal microbiota generated by principal component analysis, which were used in the analysis of covariance as covariates.

	Principal components of intestinal microbiota	
	PC-3	PC-4
Contribution of components (%)	42.2	35.1
Factor loading		
Bifidobacterium	0.853	-0.294
Bacteroides	-0.675	-0.666
Lactobacillales (Order)	0.627	0.173
Clostridium subcluster XIVa	-0.334	0.920

Abbreviations: PC principal components

Supplemental Table 3. Factor loadings of food items in the dietary pattern scores generated by the reduced rank regression

	DP-1	DP-2	DP-3	DP-4	DP-5
Low-fat milk and yogurt	-0.014	-0.005	-0.162	**-0.202**	-0.150
Normal or high-fat milk and yogurt	0.002	0.044	-0.197	0.122	0.080
Chicken (including minced)	0.014	0.038	-0.080	0.156	**-0.252**
Pork, beef, mutton (including minced)	0.075	0.038	**-0.243**	-0.020	0.005
Ham, sausage, bacon	-0.128	-0.148	-0.007	-0.080	0.086
Liver	0.030	0.087	-0.163	0.096	-0.123
Squid, octopus, shrimp, shellfish	0.022	0.031	-0.077	-0.132	**0.297**
Fish eaten with the bones	0.051	0.081	-0.056	-0.079	-0.167
Canned tuna	-0.084	-0.105	-0.043	0.090	0.077
Dried fish, salted fish, fish paste products	0.000	-0.005	-0.121	-0.003	0.023
Oily fish	-0.028	0.000	-0.121	-0.027	**0.215**
Less oily fish	0.042	0.066	-0.107	0.069	0.144
Eggs	0.075	0.114	**-0.211**	-0.069	0.101
Tofu, thick fried tofu, soy milk	0.120	0.168	-0.113	-0.096	**0.386**
Natto (fermented soybeans)	0.144	**0.232**	0.009	**-0.336**	0.161
All kinds of potatoes	0.123	0.100	0.012	-0.098	**0.269**
Pickled dark green leafy vegetables	0.097	0.059	0.095	-0.074	0.009
Other pickles	0.083	0.039	0.062	0.071	-0.037
Salad (excluding tomatoes)	0.198	0.232	0.027	**0.230**	**-0.236**
Dark green leafy vegetables, broccoli, bitter gourd	**0.376**	**0.396**	-0.109	0.123	**-0.283**
Cabbage, Chinese cabbage	**0.334**	**0.312**	0.039	**0.233**	**-0.236**
Carrots, pumpkin	**0.358**	**0.330**	-0.021	0.055	-0.076
Radish, turnip	**0.252**	**0.243**	-0.090	-0.158	0.021
Other root vegetables	**0.328**	**0.307**	0.034	0.092	0.008
Tomatoes, tomato ketchup, stewed tomatoes	0.138	0.108	-0.036	-0.093	-0.050
All kinds of mushrooms	**0.221**	0.166	**-0.225**	-0.005	0.041
All kinds of seaweed	0.079	0.061	-0.079	-0.029	-0.044
Western confectionery, cookies, biscuits	-0.136	-0.106	**-0.265**	0.118	0.049
Japanese confectionery	-0.050	-0.060	-0.156	0.028	-0.032
Rice crackers, snacks, rice cakes, Japanese pancakes, etc.	0.195	0.139	0.130	0.039	-0.053
Ice cream	-0.132	-0.131	-0.185	0.063	0.103
Citrus fruits	0.188	0.152	-0.147	0.023	-0.088
Persimmons, strawberries, kiwifruits	0.041	0.058	-0.071	0.009	0.025
Other fruits	0.016	-0.021	-0.096	-0.153	-0.038
Mayonnaise, dressing	0.084	0.089	-0.090	0.115	-0.121
Bread (including side dish bread and sweet bread)	-0.044	-0.052	-0.082	-0.056	-0.026
Buckwheat noodles	0.096	0.068	0.061	-0.002	0.064
Japanese wheet noodles	-0.041	-0.116	0.020	0.055	-0.055
Chinese noodles (including instant noodles)	-0.142	-0.199	-0.033	-0.004	-0.115
Spaghetti, macaroni	0.120	0.073	-0.071	-0.096	-0.142
Green tea	0.010	0.091	0.075	**0.523**	0.132
Black tea, oolong tea	0.013	0.036	-0.103	**0.344**	0.010
Coffee	0.052	0.043	-0.100	-0.196	-0.095
Cola, soft drinks	-0.037	-0.045	-0.113	-0.024	-0.057
100% fruit juice	-0.082	-0.083	0.051	-0.123	-0.045
Sugar	-0.154	-0.142	0.009	0.024	0.062
Rice	-0.096	-0.090	**0.490**	-0.013	-0.035
Miso soup	0.092	0.090	**0.283**	0.014	0.179
Rice wine	-0.011	-0.014	-0.149	-0.079	-0.081
Beer	0.069	0.099	-0.050	-0.053	0.011
Distilled spirit	-0.002	-0.009	-0.184	-0.110	**-0.241**
Whiskey	-0.001	0.022	-0.065	-0.006	-0.126
Wine	0.019	-0.027	-0.092	-0.103	0.007

Abbreviations: DP dietary pattern

Supplemental Table 4. Comparison of fecal bile acid levels between tertiles according to dietary pattern 1 scores

	n	Crude		Adjusted *	
		Geometric mean	95% CI	Geometric mean	95% CI
Total bile acids (μmol/g) †					
Tertile 1 (low)	21	3.71	(2.70, 5.00)	3.75	(2.71, 5.11)
Tertile 2	21	3.27	(2.36, 4.43)	3.22	(2.30, 4.38)
Tertile 3 (high)	21	3.31	(2.38, 4.48)	3.32	(2.38, 4.51)
p		0.818		0.776	
CA (μmol/g) †					
Tertile 1 (low)	21	0.11 [a]	(0.00, 0.38)	0.17	(0.00, 0.43)
Tertile 2	21	0.56 [ab]	(0.26, 0.93)	0.51	(0.24, 0.83)
Tertile 3 (high)	21	0.66 [b]	(0.34, 1.06)	0.63	(0.34, 0.97)
p		0.024		0.059	
CDCA (μmol/g) †					
Tertile 1 (low)	21	0.08 [a]	(0.00, 0.27)	0.11	(0.00, 0.32)
Tertile 2	21	0.32 [ab]	(0.12, 0.56)	0.29	(0.10, 0.51)
Tertile 3 (high)	21	0.50 [b]	(0.26, 0.77)	0.48	(0.26, 0.74)
p		0.025		0.062	
DCA (μmol/g) †					
Tertile 1 (low)	21	1.87 [a]	(1.31, 2.57)	–	
Tertile 2	21	1.14 [ab]	(0.73, 1.67)	–	
Tertile 3 (high)	21	0.92 [b]	(0.54, 1.38)	–	
p		0.031		– ‡	
LCA (μmol/g) †					
Tertile 1 (low)	21	1.31 [a]	(0.94, 1.74)	1.20 [a]	(0.85, 1.61)
Tertile 2	21	0.51 [b]	(0.27, 0.79)	0.54 [b]	(0.30, 0.82)
Tertile 3 (high)	21	0.41 [b]	(0.19, 0.67)	0.45 [b]	(0.23, 0.72)
p		< .001		0.003	

* Adjusted for defecation status (Two principal components generated from the frequency of hard, normal, and watery stools by principal component analysis) and intestinal microbiota (Two principal components generated from the *Bifidobacterium*, *Lactobacillales*, *Bacteroides*, and *Clostridium* subcluster XIVa by principal component analysis).

† Bile acid levels were measured per fresh fecal mass.

abc Different letters indicate statistically significant differences between the groups (Sidak post hoc test, $p < 0.05$).

‡ An ANCOVA could not be used to assess differences due to significant interaction.

Abbreviations: CI, confidence interval; CA, cholic acid; CDCA, chenodeoxycholic acid; DCA, deoxycholic acid; LCA, lithocholic acid

Supplemental Table 5. Comparison of fecal bile acid levels between tertiles according to dietary pattern 3 scores

	n	Crude		Adjusted *	
		Geometric mean	95% CI	Geometric mean	95% CI
Total bile acids (μmol/g) †					
Tertile 1 (low)	21	4.60 [a]	(3.45, 6.03)	4.52	(3.37, 5.97)
Tertile 2	21	3.17 [ab]	(2.32, 4.25)	3.17	(2.30, 4.27)
Tertile 3 (high)	21	2.71 [b]	(1.95, 3.67)	2.76	(1.98, 3.74)
p		0.039		0.065	
CA (μmol/g) †					
Tertile 1 (low)	21	0.54	(0.24, 0.92)	0.53 [ab]	(0.26, 0.85)
Tertile 2	21	0.18	(0.00, 0.47)	0.16 [a]	(0.00, 0.41)
Tertile 3 (high)	21	0.58	(0.27, 0.97)	0.62 [b]	(0.34, 0.95)
p		0.125		0.038	
CDCA (μmol/g) †					
Tertile 1 (low)	21	0.47 [a]	(0.24, 0.74)	0.47 [a]	(0.25, 0.72)
Tertile 2	21	0.09 [b]	(0.00, 0.29)	0.08 [b]	(0.00, 0.27)
Tertile 3 (high)	21	0.32 [ab]	(0.12, 0.57)	0.34 [ab]	(0.14, 0.57)
p		0.049		0.029	
DCA (μmol/g) †					
Tertile 1 (low)	21	1.60 [a]	(1.10, 2.22)	1.57 [a]	(1.07, 2.19)
Tertile 2	21	1.61 [a]	(1.11, 2.24)	1.66 [a]	(1.14, 2.30)
Tertile 3 (high)	21	0.74 [b]	(0.40, 1.15)	0.73 [b]	(0.39, 1.14)
p		0.012		0.011	
LCA (μmol/g) †					
Tertile 1 (low)	21	0.79 [ab]	(0.49, 1.17)	0.77 [ab]	(0.49, 1.11)
Tertile 2	21	0.95 [a]	(0.61, 1.35)	0.98 [a]	(0.67, 1.36)
Tertile 3 (high)	21	0.40 [b]	(0.16, 0.69)	0.40 [b]	(0.17, 0.66)
p		0.041		0.019	

* Adjusted for defecation status (Two principal components generated from the frequency of hard, normal, and watery stools by principal component analysis) and gut microbiota (Two principal components generated from the *Bifidobacterium*, *Lactobacillales*, *Bacteroides*, and *Clostridium* subcluster XIVa by principal component analysis).
† Bile acid levels were measured per fresh fecal mass.
[abc] Different letters indicate statistically significant differences between the groups (Sidak post-hoc test, $p < 0.05$).
Abbreviations: CI, confidence interval; CA, cholic acid; CDCA, chenodeoxycholic acid; DCA, deoxycholic acid; LCA, lithocholic acid
